# Antimicrobial, antioxidant and anti-inflammatory activities of seeds from *emblica officinalis* (Gaertn.)

**DOI:** 10.6026/97320630018683

**Published:** 2022-08-31

**Authors:** Pooja G Singh, Anisha S Jain, Poojitha B Sridhara Setty, Sushma BV, Sharanagouda S Patil, Ashwini P, Gopenath TS, Kuralayanapalya Puttahonnappa Suresh, Guru Kumar Dugganaboyana, Karthikeyan Murugesan, Ashok Gnanasekaran, Chandan Shivamallu, Shiva Prasad Kollur, Chandrashekar Srinivasa, Raghavendra HL, Parthiban Rudrapathy, Kanthesh M Basalingappa

**Affiliations:** 1 JSS Academy of Higher Education and Research, Mysuru, Karnataka 570 015, India; 2 Department of Studies in Biotechnology, Davangere University, Davangere, India; 3 ICAR-National Institute of Veterinary Epidemiology and Disease Informatics, Yelahanka, Bengaluru, Karnataka 560 064, India; 4 Department of Microbiology, Faculty of Medicine, Quest International University, Ipoh, Perak, Malaysia; 5 Department of Sciences, Amrita School of Arts and Sciences, Amrita Vishwa Vidyapeetham, Mysuru Campus, Mysuru, Karnataka 570 026, India; 6 Center for Biotechnology, Pravara Institute of Medical Sciences, Loni-413736, Rahata Taluk, Ahmednagar District, Maharashtra, India; 7 Malabar Cancer Centre, Department of Clinical Laboratory Services & Translational Research, Thalassery, Kannur 670103, India

**Keywords:** Emblica officinalis, antioxidant, lipoxygenase inhibition, antimicrobial, LC-MS analysis

## Abstract

There is a shred of evidence to suggest that Emblica officinalis Gaertn, the botanical name for amla seeds, has greater medicinal potential than amla fruit. We conducted this work to assess the anti-inflammatory, antibacterial, and antioxidant capacities of E. officinalis seed extracts. The bioactive components from the seeds were fractionated using chloroform, hexane, methanol, and diethyl ether, according to the polarity of the solvents. The total amount of phenolic and flavonoid was estimated. Both the reducing power and antioxidant capacities of the extracts were evaluated using the DPPH (1,1-diphenyl-2-picryl-hydrazyl) technique. 15-lipoxygenase (LOX) was inhibited by seed extracts at doses ranging from 5 to 25 micrograms. In silico docking was employed to assess the results. Some human pathogenic microorganisms were tested for their antibacterial activity using the agar disc diffusion method. Escherichia coli, Proteus vulgaris, and Klebsiella pneumonia were inhibited by a methanolic extract with an IC50 value of 58g, making it the most common organic solvent extract. Methanolic extracts also showed good antioxidant and antibacterial activity. Our investigation led us to discover that amla seeds have anti-inflammatory, antioxidant, and antibacterial effects.

## Background:

Most of the human diet is comprised of herbs and spices. Their nutritional, medicinal and preservative properties are well-known in addition to their flavor [1]. Emblica officinalis' anti-microbial properties and lack of side effects have made it more popular in recent years, despite its long recovery time [2]. Medicinal plant E. officinalis Gaertn is widely used in Ayurvedic and Unani medicine systems [3]. The sour amla fruit is often soaked in saltwater and turmeric to make it more palatable in India or, in other words, pickled [4]. The amla fruit's chemical composition contains more than 80% water [5]. Protein, carbohydrate, fibre, and mineral content are also present, as well as gallic acid, which is a highly effective polyphenol [6]. When it comes to the physical and chemical properties of the fixed oil yield (16%), Iodine 139.5, unsaponifiable matter 381.8% and saturated fatty acids 72% are found in the linolenic acid (8.78%), the linoleic acid (44 %), and the oleic acid (28.10%) (0.95%) [7-8]. Amla is the most concentrated source of Vitamin C in the plant kingdom. It is easily absorbed by the human body when the entire fruit is used instead of an active ingredient. The tannins in the amla fruit bind to the vitamin C present in the fruit showing anti-diarrheal and antipyretic properties. E.officinalis contains several chemical constituents. Microbe-fighting constituents include quercetin and flavonoids (phyllantine, phyllantidine), gallic acid, ascorbic acid and hydrolyzable tannins (emblicanin A and B). Therefore, it is of interest to evaluate the antioxidant, anti-inflammatory and antibacterial activity of different solvent extracts of E.officinalis seeds.

## Materials and Methods:

Reagents and Chemicals Catechin, 1,1-diphenyl-2-picryl-hydrazyl (DPPH) and linoleic acid were procured from Sigma-Aldrich. Folin-Ciocalteu reagent, gallic acid, ascorbic acid, trichloroacetic acid, ferric chloride, aluminum trichloride, sodium carbonate and sodium hydroxide and other reagents and chemicals used were of AR grade.

### Plant Extract:

#### Seeds and rind:

 Fresh fruits of amla (Emblica officinalis) were chosen from the local supermarket in Karnataka State, India. Seeds and rinds were segregated from the fruit and shade dried at 37°C for 3 days. The dried samples were coarsely ground using 500 ml of liquid nitrogen in a mortar and pestle. As soon as the amla seeds and rinds were ground to a coarse paste, they were stored in sealed glass bottles at 4°C until they were used [9] [10].

### Extraction of seeds and rinds: 

On the basis of increasing polarity, hexane, chloroform, diethyl ether, and methanol solvents were used to dissolve 25 g of powdered amla seeds in 200 ml of solvent. The extraction was done for 48 hours at room temperature with a rotator shaker set to 30°C and 60 rpm. Filtration of the solvent via muslin cloth was used to separate it. The samples were then vacuum filtered and dried in glass petri plates at room temperature using a flash evaporator set to 44°C and 160 rpm. The final drying procedure was carried out for 3 hours at 40°C using a Speed Vac Concentrator (Savant SPD 2010), and the extracted material was kept in the dark at 4°C until further usage [11].

### Quantitative Analysis of Phytochemical Constituents: 

#### Determination of total phenolics: 

The Folin-Ciocalteu colorimetric technique was used to calculate the total phenolic content. The test sample (100 µl) was incubated at 22°C for 5 minutes with 250 µl of Folin-Ciocalteu reagent. The reaction was neutralized with 1500 µl of saturated sodium carbonate and left to sit in the dark for 1.5 hours at 22°C. A Hitachi U-3900 UV/visible spectrophotometer were used to test the blue color's absorption at 765 nm. A calibration curve based on the absorbance of known amounts of Gallic acid standard (20 to 100 µg) was used to calculate total phenolics. Gallic acid equivalence (GAE) in µg was used to calculate the total phenolic content [12].

###  Estimation of total flavonoids: 

The total flavonoid content was estimated using aluminum trichloride by the colorimetric method. Catechin (1–5 µg) was utilized as a control. Extract (10-50 µl) was made up to 1 ml using methanol, 4 ml distilled water, and 0.3 ml of 5% sodium nitrite solution. After 5 minutes of incubation, 0.3 ml of 10% aluminum trichloride solution was added and the combination was allowed to stand for 6 minutes. The combination was then raised to a final volume of 20ml with double-distilled water after the addition of 2ml of 1mol/L NaOH solution. The absorbance was measured at 510 nm after the mixture had been allowed to sit for 15 minutes. The total flavonoids content was determined using a calibration curve observed from measuring the absorbance of known concentrations of catechin as standard (10 to 50µg). The total flavonoid content was expressed as catechin equivalence (CE) in µg [13].

### Test for tannins: 

Around 0.1 g of amla seed extract was weighed and suspended in respective solvents. In a test tube, diluted extract (0.5 mL) was mixed with 10 mL distilled water and filtered using Whatman No.1 filter paper. Three drops of ferric chloride reagent were applied to the filtrate. The existence of gallic or catechol tannins was established by the emergence of blue-black or green precipitation [14].

###  Test for Saponins: 

01. g of amla seed extract was weighed and diluted in respective solvents. In test tubes, different solvent extracts (1 mL) were added to 5 mL of distilled water. The presence of saponins was shown by the formation of stable foam.

### Estimation of antioxidant activity: 

DPPH radical scavenging activity An amla seed extract in hexane, diethyl ether, and methanol was tested for its ability to neutralize free radicals using the DPPH method. A solution of DPPH radicals was made in methanol [15]. This mixture comprises 5 µl of test samples and 95 µl of 50 µg/ml DPPH. After 30 minutes at 37°C in the dark, a 96-well plate was used to perform the DPPH radical scavenging reaction. The radical scavenging activity was determined as a percentage using a solvent as a control [16]. The IC50 values were calculated, which show the concentration of extracts required to scavenge 50% of DPPH radicals. The standard was ascorbic acid (1-10 µg). The following equation was used to calculate the percent scavenging effect:

Eq. (A.1).Inhibition (%) = [(Absorbance of control -Absorbance of the test sample)]/Absorbance of control x 100 

### Reducing power estimation: 

The reducing power assay of amla seed extract was determined by ferric chloride (FeCl3). Phosphate buffer (0.2 M, pH 6.6) and potassium ferricyanide were combined in equal parts with the test sample (0.1 g) (2.5 ml, 1%). It was incubated for 20 minutes at 50°C. The mixture was then centrifuged at 3000 rpm for 5 minutes with trichloroacetic acid (TCA) (10 percent, 2.5 ml). The supernatant (1 ml) was collected after centrifugation and combined with an equal volume of distilled water and FeCl3 in a test tube (0.5 ml, 0.1%). As a blank solution, phosphate buffer was utilized, and ascorbic acid was employed as a standard. Beckman-DU730 Coulter's life sciences UV/Visible spectrophotometer was used to detect absorbance at 700 nm. Stronger reducing power is shown by increased absorbance of the reaction mixture.

### Anti-inflammatory Activity: 

#### In vitro activity: 

The anti-inflammatory property was determined spectrophotometrically [17] with a slight modification in the procedure. Lipoxygenase (LOX) enzyme activity was assayed in a spectrophotometer. The 15-lipoxygenase (15-LOX) from soybeans was used in the test. Experimentally, the loss of soybean 15-LOX activity (5 g) was measured by using 0.2M linoleic acid (Sigma) as the substrate prepared in the solubilized state (dissolving in 10 µl methanol) in the presence of 0.2M borate buffer (pH 9.0) [18]. With a UV-Vis spectrophotometer, various extract concentrations (5, 10, 15, 20 and 25 µg) and a reference compound (ascorbic acid) was used to measure inhibition. We also calculated the percentage of enzyme activity that the extracts inhibited. IC50, or the concentration needed to inhibit 50% of LOX activity, was estimated as well [19]. The following equation was used to calculate hydrogen peroxide content and lipoxygenase activity:

Eq. (A.2). Specific activity (LOX)= delta A.V/tau.l.c

Where delta A is the absorbance rise per minute, V is the incubation mixture volume, tau is the extinction coefficient for linoleic acid (25 x 10-3 mol/l/cm) [20], l is the length of the cuvette (1 cm) and c is the enzyme concentration in mg (0.005). The results represent the average of three separate tests.

### Molecular docking: 

A molecular docking approach is used to determine the interactions and binding affinity between three-dimensional structures of a ligand and protein. To carry out this, PyRx 0.8 virtual screening software was used. The 3D structure of the target protein, soybean 15-lipoxygenase (15-LOX) was retrieved from Protein Data Bank (PDB) having a resolution of 2.63 Å [22]. To prepare the target protein for docking, water molecules and bound ligands were detached from the structure, missing residues and polar hydrogens were added to it (Figure 1 - see PDF). Among several phytocompounds present in the seed extract of E.officinalis [22], [23] we selected a few compounds, whose 3D structures were downloaded from the PubChem database. These structures were prepared for docking by converting the downloaded sdf files into pdb files using Open Babel GUI. The binding site of the target protein was estimated by CASTp online server. Along with the binding residues, the area and volume of the binding pocket are also calculated by this server. Finally, the protein and ligand files are loaded to the PyRx server where the docking analysis is carried out [24].

### Antibacterial Activity: 

Antibacterial activity of seed extracts was tested against gram-negative bacteria Klebsiella pneumonia and Escherichia coli and gram-positive bacteria Pseudomonas vulgaris which were collected from the Department of Microbiology, JSS Academy of Higher Education and Research, Mysuru, India. All of these cultures were kept at 4°C on nutrient agar plates.

### Agar disc diffusion: 

To test for antibacterial activity, methanolic seed extract of E.officinalis was tested against a bacterial culture. Muller Hinton agar was inoculated evenly with 0.02 ml of a 24hr old culture using a sterile cotton swab. This created a lawn culture on the MHA. A seeded agar plate was used to test the antibacterial activity of methanolic extract of E.officinalis. The discs containing the extract were placed on the seeded agar plate [25], [26]. Discs carrying methanol solvent were used as controls. Streptomycin (10 µg) antibiotic was used as a drug. Varying concentrations of methanol extract (1, 2.5, 5, 10 µg) were used. The plates were incubated at room temperature for 48 hours before being examined for a zone of growth inhibition around the discs (Stereo Discovery V20 - Zeiss, Germany, Whitefield Microscope). The antibacterial activity of the extracts was determined by measuring the diameter of the zone of inhibition to the nearest millimeter for each bacterial species.

### LC-MS analysis: 

According to the manufacturer's protocol, the metabolites were analyzed qualitatively using Synapt G2 (UPLC separations with Quan Tof). The nitric oxide flow rate was set at 10 L/min at 350°C with a 60-psi nebulizer pressure. Positive ionization was used to acquire mass spectra from m/z 100 to 1000. For fragmentation of the isolated compounds, helium was used as a collision gas [27]. The detection conditions were as follows: skimmer voltage, -40 V; capillary voltage, 3500 V; trap drive level, 45.0; Oct RF, 150 Vpp; Lens 1, 5.0 V; Lens 2, 60 V; cap exit voltage, -158.5 V; Oct 1 DC, -12 V; Oct 2 DC, -2.45 V.

## Results:

### Phytochemical constituents and active ingredients: 

The physical properties of pH and moisture content of amla were found to be slightly acidic and 65% respectively. The Phytochemicals analysis of amla seed extract is summarized in Table 1 (see PDF). Amla seeds contain a lot of nutritive and anti-nutritive compounds. Saponins, tannins, flavonoids and phenolic content were `found to be higher in methanolic extract than other solvent extraction. Smaller amounts of the biologically active compound were extracted by hexane, diethyl ether and chloroform. The amount of total phenol was observed as 120 µg (Figure 2 - see PDF) of seeds as gallic acid equivalents and the amount of total flavonoids content was observed as 100 µg (Figure 3 - see PDF) as catechin equivalents of amla seeds.

### DPPH free radical scavenging activity and reducing power assay: 

The antioxidant activities of E.officinalis seed extracts were confirmed in our work using the DPPH and Reducing Power Assays. The hydrogen donating ability of test compounds is ascribed to the percentage of DPPH decolonization. The amla seed extract showed a wide range of DPPH activity. When compared to other extracts, Methanolic extract had higher antioxidant activity (IC5024 µg), whereas Hexane (Figure 5 - see PDF) and Diethyl ether (Figure 6 - see PDF) had the lowest DPPH radical scavenging activity. At 42 µg, the reference standard ascorbic acid demonstrated 50% inhibition.

### Reducing power estimation: 

The IC50 of the methanolic extract is 40µg in the reducing Power assay (Figure 7 - see PDF). The methanol extracts of amla seed had good activity, whereas hexane and diethyl ether exhibited modest activity. Ascorbic acid, the reference standard, demonstrated a 50% inhibition. Lower IC50 value implies higher antioxidant power. The presence of reductions capable of breaking free radical chains by donating hydrogen atoms and therefore converting them to a more stable non-reactive species is indicated by a higher reducing power.

Lipoxygenase inhibition Hydroperoxylinoleic acid was detected as a rise in absorbance at 234 nm, indicating LOX activity. Concentration-dependent LOX inhibition was observed with E.officinalis seed extract. The methanolic extract had the strongest inhibitory effect, with an IC50 of 58 µg. At a dose of 100 µg, complete LOX inhibition was found (Figure 8 - see PDF).

### Molecular docking analysis 

The docking analysis was carried out between the target protein of 15-lipoxygenase and bioactive compounds of Emblica officinalis. Table 2 and Figure 9 (see PDF) represent the estimated binding affinity of the best-docked poses obtained from PyRx. An acceptable binding affinity between protein and ligand is indicated by low binding energy. Based on the graph, we can say that quercetin has shown the best binding affinity (-9.7 Kcal/mol) with the active site of the target protein followed by gallocatechin (-9.1 Kcal/mol). These docked complexes were further evaluated using visualization software. The docked complexes of 15-LOX with 2 two best compounds that are, quercetin and gallocatechin were further evaluated using Chimera 1.15 and the 2D images were procured from BIOVIA Discovery Studio Visualizer 2020. Both the ligands showed a high number of non-bonded interactions with the binding site residues of the target protein. In Figure 10 (See PDF), we can see that quercetin shared five hydrogen bonds with TYR A: 107, GLN A: 108, ARG A: 145, HIS A: 394, PHE A: 399 (bright green colored discs and bonds), a pi-sigma bond with ILE A: 403 (purple colored disc and bond) and fourteen van der Waals interactions (light green discs). An unfavorable bond was also observed with THR A: 406 (red-colored disc). For the docked complex of gallocatechin (Figure 11 and Figure 14 - see PDF), there were four hydrogen bonds with HIS A: 394, ARG A: 145, TYR A: 149, ARG A: 407, a single pi-sigma bond with ILE A: 403, an unfavorable bond with GLN A:108 and eleven van der Waals interactions.

### Antibacterial activity: 

As an antipyretic, analgesic and immunomodulatory agent, E.officinalis Gaertn has a long history of use. First, the antibacterial property of the extracts was determined using the standard agar disc diffusion method, as previously reported [28]. Extracted seed extracts were compared to streptomycin, a commonly used antibacterial drug, for their antibacterial efficacy in vitro. The methanolic solvent extract of the seed showed antibacterial property against the clinical cultures of E. coli, P. vulgaris and K. pneumonia and the results were recorded at different time intervals of 12h, 24 h and 48h and the results for E. coli is presented in Figure 12 (see PDF). The results for P. vulgaris are presented in Figure 13 - see PDF. Maximum antibacterial activity was obtained for E. coli (4.32 mm); (Table 3 - see PDF) and minimum response was recorded against P. vulgaris for methanolic extracts (Table 4 - see PDF). No activity was recorded for K. pneumonia.

### LC-MS estimation: 

The methanol seed extract of E. officinalis yielded 6 compounds. The mass spectral analysis was used to make a tentative identification of these compounds, which was then confirmed by comparing them to literature values. Complicated metabolite identification using chromatographic and internal MS data identified compound A as MS1-Digalloyl Hexose and Diaglloyl Glucose isomers. Compound B was identified as Patuletin glucoside and Patuletin Gallacatechin. The compound C is identified as Methyl 4,7,10,13,16 and 19-doco sahenaenoate (C23H34O2), Compound D was identified as MS3 cis-resveratrol, caftaric acid and Quercitrin and Compound E were identified as MS4- (±) - naringenin by comparing retention time and MS/MS data (Figure 14 - see PDF).

## Discussion:

A sophisticated traditional medicine system is based on plants, and natural products are excellent starting points for novel drug development programs [29]. Health programs in developing countries can benefit from the effective use of herbal medicine that the World Health Organization (WHO) encourages, promotes, and facilitates [30]. Studies have been conducted to determine the potential of natural products in inhibiting ROS and, as a result, attenuating health situations requiring ROS inhibition. It has been three decades since new antibiotics were created, yet bacteria have become more resistant to them [31]. Drug resistance is a common trait among bacteria. In the present study, a freshly prepared DPPH solution with maximum absorption at 517 nm is used in the DPPH method. Most of the time, if there is an anti-oxidant present in the medium, the purple color fade [32]. Antioxidant molecules provide hydrogen or electrons to DPPH free radicals, perhaps via a free radical attack on the DPPH molecule resulting in an increase in absorbance at 517nm. As a result, the extract's antioxidant potency increases as the absorbance decreases. Indicators of potential antioxidant activity include a compound's reducing capacity [33]-[34]. Most of the time, the presence of reductions, which break the free radical chain by giving away a hydrogen atom, is linked to the reducing ability. The extract showed a reductive ability that grew when the concentration of the extract was raised [23]. An antimicrobial activity screening of plant extracts revealed that higher plants are a potential source of new anti-infectives. Although conventional antibiotics inhibit bacteria through different mechanisms, plant-derived antimicrobial compounds may have clinical value in treating infections caused by resistant microbes. Gram (-) bacteria, such as *E. coli*, and Gram (+) bacteria, such as P. vulgaris, were the most susceptible to methanol extract Differential susceptibility of Gram-positive or Gram-negative bacteria to phenolics was previously reported [35]. A major role is played by inhibiting the production of leukotrienes (LTs) and PGE2. Inflammation-inducing enzymes are inhibited in the initial screening of plants used for hydroxy peroxy linoleic acid is formed when the LOX activity increases. They provide valuable preliminary data for selecting seed extract with potential anti-inflammatory activities that support their traditional use. Methanolic seed extract with potent LOX inhibition capacity [36]. Antioxidant activity has been related to plant phytochemicals, which humans cannot synthesize [13]. In the current study, UPLC-DAD identified cis-resveratrol, gallo catechin, quercetin among others. Gallocatechin and quercetin have also proved to be effective enough against the LOX protein via in silico analysis. According to Indian traditional literature, the presence of phytochemicals in the extract of amla seeds may also contribute to their medicinal qualities.

## Conclusions:

With this study, we wanted to see how seed extracts of E. officinalis Gaertn worked as anti-oxidants, anti-inflammatory agents, and anti-microbial agents in vitro Hexane, chloroform, diethyl ether, and methanol solvents were used to separate the bioactive components from the seeds in increasing order of polarity. They were tested for antioxidant properties using the DPPH (1,1-diphenyl-2-picrylhydrazyl) method, and for reducing power using the FeCl3 method, as well. Seed extracts were used to inhibit 15-lipoxygenase (LOX) at concentrations ranging from 5 to 25µl. A good antioxidant and antibacterial activity were observed in the methanol extracts, which were the most common organic solvent extract inhibition of Lox by methanolic extract with an IC50 value of 58µg have been reported. Total antioxidant capacities were found to be positively correlated with phenolic content, indicating that the phenolics in amla seeds were the dominant antioxidant constituents. We can confirm that amla seeds contain anti-oxidant, anti-inflammatory, and antibacterial activities as a result of our research.
